# Discovery of Novel Animal-Based Medicinal Products with Therapeutic Potential in Evidence-Based Traditional Medicine

**DOI:** 10.1155/2019/1626543

**Published:** 2019-12-19

**Authors:** Gunhyuk Park, Yong-ung Kim, Irawan W. Kusuma

**Affiliations:** ^1^Herbal Medicine Reseources Research Center, Korea Institute of Oriental Medicine, 111 Geonjae-ro, Naju-si, Jeollanam-do 58245, Republic of Korea; ^2^Department of Pharmaceutical Engineering, College of Biomedical Science, Daegu Haany University, Gyeongsan, Republic of Korea; ^3^Forest Products Chemistry Laboratory, Faculty of Forestry, Mulawarman University, Samarinda, Indonesia

Animals have been used as traditional medicinal resources for the treatment and relieve of a myriad of illnesses and diseases in practically every human culture. The traditional use of animals and their products for medicinal purposes has been documented since ancient times in civilizations from East Asia and Africa. In China, more than 1500 animals are used as medicine; in India, 15–20% of Ayurvedic medicine is based on animal-derived substances, whereas in Latin America, 584 medicinal animal species have been recorded. Ho Jun, a Korean court physician (1610 AD), wrote “Dongui-Bogam,” which contains references to nearly 95 insects only and its substances. Recently, as such, there is an active study of the animal-based medicine. Animal-based medicines have been elaborated from parts of the animal body, from products of its metabolism (corporal secretions and excrements), or from nonanimal materials (nests and cocoons). Also, recently insects have proven to be very important as sources of drugs for modern medicine since they have immunological, analgesic, antibacterial, diuretic, anesthetic, and antirheumatic properties. Animals have been methodically tested by pharmaceutical companies as sources of drugs to the modern medical science. Regarding fish, several compounds have been extracted and these are employed as remedies in the official medicine. Toxins in snake, spider, centipede, and other species' venom have shown promise for treating chronic pain, heart conditions, blood clots, and type 2 diabetes. However, many animal medicines have not yet been tested for efficacy. To scale up production of such medicines at an industrial level, it is important to lay the scientific foundation to recognize them as potential therapeutics and to provide insights into their pharmacological activity and other relevant aspects; these studies may pave the way to develop new drugs that would potentially alleviate human suffering. Thus, new pharmacological therapeutic strategies, involving the use of animal-based medicinal products or compositions of multiple extracts, are being designed to specially act on biochemical targets.

In this special issue, investigators contribute original research articles and review articles that would facilitate the understanding of the basic mechanisms as well as the development of new and promising complementary and alternative strategies for the potential of animal-based medicinal products.

Y. Zhu et al. reported the seizures of chronic epilepsy rats injected by pentylenetetrazol-kindled through inhibiting the abnormal discharge of hippocampal neurons in epileptic rats. They suggested that treatment of epilepsy pill can effectively improve the spatial learning and memory ability of the epilepsy model. Also, the dingxian pills might interfere with epilepsy as well as cognitive dysfunction through the Egr3-GABRA4 signal, NRG1-ErbB4 signal, and ERK-Arc pathway. Thus, this may be a candidate for the treatment of convulsant.

H.-S. Lim et al. reported potential subacute toxicity of Mantidis Ootheca water extract during a 2-week repeated oral administration of doses of 0, 50, 150, or 450 mg/kg/day to C57BL/6 male mice by gavage. They suggested a 14-day repeated oral dosing of Mantidis Ootheca extract to mice firstly resulted in a significant alteration in clinical signs, body weight, and hematological, biochemical, and histopathological parameters at doses of ≤450 mg/kg/day. The authors have judged that the Mantidis Ootheca is safe up to 450 mg/kg and is helpful in further development.

Then, T. Wang et al. confirmed the validation and stability of concentration analysis method of the Mecasin (Gamijakyakgamchobuja-Tang) preparations using high-performance liquid chromatography. They suggested that preparations at concentrations of 50 mg/ml and 200 mg/ml in sterilized distilled water were homogeneous and stable for 4 hours at room temperature and for 7 days in refrigerated conditions. This method for analyzing Mecasin preparations was deemed suitable. Additionally, this study promotes the development of reliable manufactured medicines and conduct of good research through definitive quality controls for Mecasin as a complex herbal medicine, with the aim of treating amyotrophic lateral sclerosis.

J. Tan et al. investigated the methanolic extract of *Aspongopus chinensis* can inhibit the proliferation of MDA-MB-453 and HCC-1937 cells by inducing cell cycle arrest; the oleic acid and palmitic acid were found to be the main compounds in *Aspongopus chinensis*. Their results suggested that *Aspongopus chinensis* can be a potential natural alternative or can provide a complementary therapy for breast cancer.

D. A. Putri et al. reported bioactive compounds in *Chromolaena odorata* with reactive oxygen species scavenging activity. Among the tested five extracts, the ethyl acetate extract exhibited the highest inhibitory effect against ABTS radical and *α*-glucosidase rat intestinal enzyme. Further investigations focus on the identification of the other active flavanone compounds responsible for the antioxidant as well as *α*-glucosidase inhibitory activity of *Chromolaena odorata* ethyl acetate extract. So, these beneficial active ingredients are thought to be able to be developed as a growth factor for insect breeding farms in the future.

D. Lee et al. reported that musk of muskrat protects neurons against focal cerebral ischemia in rats with functional restoration. In relation to the immunohistochemical studies, the effects of musk of muskrat may be due to their anti-inflammatory properties by inhibiting Cox-2 expression. Based on these findings, it is tempting to suppose that musk of muskrat could be considered as a substitute for musk of musk deer in view of the traditional use of stroke treatment. The authors concluded musk of muskrat a candidate for the treatment of focal cerebral ischemia.

T. Jiang et al. reported that *Scolopendra subspinipes mutilans* treatment improved mean arterial pressure, heart rate, central venous pressure, fatigue, palpitation, and shortness breath in the coronary heart disease patients. Meanwhile, *Scolopendra subspinipes mutilans* intervention reduced the levels of blood lactate, creatine kinase isoenzyme, serum troponin T, CRP, IL-1*β*, IL-6, and TNF-*α*, and malondialdehyde and increased superoxide dismutase level. Also, *Scolopendra subspinipes mutilans* treatment reduced the levels of IL-1*β* and IL-6 and increased the levels of IL-10 and TGF-*β*. *Scolopendra subspinipes mutilans* had better anti-inflammatory effects. *Scolopendra subspinipes mutilans* treatment increased the percentage of CD4 + CD25 + FoxP3 Treg cells and reduced the percentage of CD4 + IL-17 T cells. *Scolopendra subspinipes mutilans* ameliorated rheumatic heart disease by affecting the percentage of CD4 + CD25 + FoxP3 Treg and CD4 + IL17 T cells. Thus, this study may provide a new molecular mechanism for rheumatic heart disease treatment.

B. Gong et al. investigated the safety and efficacy of the Herbal Medicine C-117 formula in the treatment of carotid atherosclerosis-vulnerable plaques. After 180 days of medication, the plaque Crouse scores of the two groups were lower than before, and the total cholesterol and blood lipids decreased compared with those before treatment. The change of the C-117 formula group was statistically significant, but no significant differences were found when comparing groups after treatment. Although adverse events occurred during the 6-month treatment, there was no liver or kidney dysfunction, and all adverse events were associated with a weak correlation with the C-117 formula. Thus, they believe that the C-117 formula provided better results.

At present, the prevention and treatment of cardiovascular disease in the world are facing severe challenges. So, Xinmailong Injection, which is derived from the animal medicine Periplaneta Americana, has certain advantages in the clinical treatment of cardiovascular disease. S.-S. Lin et al. reported that Xinmailong Injection can protect cardiomyocytes and maintain the normal function of the heart in various ways, thus effectively preventing the development of cardiovascular disease. Therefore, Xinmailong Injection has great potential for clinical application, and more basic researches need to be carried out to explore the medicinal value of Xinmailong Injection.

In addition, L. Han et al. in a research article investigated that the zebrafish model provides a huge balance between scale and applicability. Based on this model, new gelatin has the protective effect on hematopoietic injury induced by chemotherapy, which is shown by its reversal effect on thrombocytopenia and erythrocyte reduction induced by chemotherapeutic drugs. Its mechanism may be related to the promotion of the expression of key hematopoietic factors.

J.-B. Zou et al. indicated that the combination of Kangfuxin liquid and proton pump inhibitors for treatment of patients with gastric ulcers could improve the total efficacy rate and that the efficacy rate and gastroscopy rate reduce adverse events and the recurrence rate. That study's importance stems from its inclusion of an extended meta-analysis and updated information, which have raised confidence in the effectiveness of Kangfuxin liquid.

We hope that the readers will be interested in animal-based medicinal products and that this special issue will attract the interest of the scientific community, thereby supporting further investigations that lead to the discovery of novel strategies and therapeutic medicinal products for use in the field of evidence-based traditional medicine.

